# Relationship between anthropometric and kinematic measures to practice velocity in elite American 100 m sprinters

**Published:** 2021-09-27

**Authors:** Amber Murphy, Kenneth P. Clark, Nicholas Murray, Bridget Melton, Ralph Mann, Randall Rieger

**Affiliations:** ^1^Department of Exercise Science, Concordia University Chicago, Chicago, USA; ^2^Department of Kinesiology, West Chester University of PA, USA; ^3^Department of Kinesiology, University of Nevada, Reno, USA; ^4^Department of Health Sciences and Kinesiology, Georgia Southern University; ^5^CompuSport, Las Vegas, NV, USA; ^6^Department of Mathematics, West Chester University of PA, USA

**Keywords:** sprint running, velocity, kinematics, elite sprinters

## Abstract

**Background:**

There exists a paucity of anthropometric and kinematic data for elite United States (US) sprinters and further analysis of how these variables correlate with sprint velocity in practice is warranted.

**Aim:**

The purpose of this investigation was to examine the relationship of anthropometric and kinematic variables and practice sprint velocity of elite sprint athletes when separated by gender.

**Methods:**

Participants included elite US 100 m sprinters (total: *n*=38, male: *n*=19, female: *n*=19). Inclusion criteria were participation in the 100 m semifinals or finals at the US Outdoor National Championships from 2015 to 2019. Anthropometric data and 300 Hz video during maximum velocity sprinting were collected during a practice session and video was digitized to determine the kinematic variables of interest. Relationships with maximal sprint velocity were assessed using Pearson’s correlation coefficient and linear regression analysis.

**Results:**

Males showed significant unadjusted relationships between practice velocity and step length (*r*=0.668; *P*=0.002), horizontal backward foot velocity at touchdown (*r*=0.459; *P*=0.048), and upper leg full extension angle (*r*=–0.585; *P*=0.009). Multiple regression analysis found that when adjusting for these three variables, step length was the only significant predictor of practice velocity in males which accounted for 44.6% of the variability in practice velocity in males. The females showed a significant relationship between practice velocity and step length (*r*=0.629; *P*=0.004) which accounted for 39.5% of the variability in practice velocity.

**Conclusion:**

These results provide researchers and coaches with important information regarding the anthropometric and kinematic variables related to elite top speed sprinting performance.

**Relevance for Patients:**

Training focused on increasing step length may be an efficient way to improve velocity in practice.

## 1. Introduction

The United States (US) is a highly competitive country in the male and female 100 m sprint events [1,2]. In the 2019 World Track and Field Championships, both gold and silver medals in the men’s 100 m event were won by members of the US team [3]. In competition, maximal velocity is a primary predictor of overall 100 m time [4]. Furthermore, prior research has indicated maximal velocity measured in an experimental testing session, laboratory or research based, is highly related to performance [5]. Therefore, for an elite population such as international level US 100 m sprinters, investigation into the anthropometric and kinematic determinants of maximal sprinting velocity in practice may be valuable in understanding sprinting performance. Maximal sprinting velocity in practice and its related variables in this population may provide insight into how these athletes generate international-level performances and thus help researchers and coaches identify optimal training strategies.

For trained sprinters, prior research has quantified the basic kinematic parameters in trained sprinters during experimental (non-competition) settings. For maximal velocity, mean values of 10.84±0.12-9.33±0.31 m/s and 9.73±0.35-8.88±0.11 m/s have been reported for males and females, respectively [5-7]. Mean contact times in both male and female sprinters typically range from 0.08 to 0.10 s, with a significant inverse relationship between contact time and maximum velocity [6,7]. Mean flight times in both male and female sprinters are typically 0.11-0.13 s, although prior research indicates no significant relationship between flight time and maximum velocity [6,7]. Mean step rates of approximately 4.4-4.95 and 4.3-4.6 steps/s have been reported for male and female sprinters, respectively, although there exists conflicting findings regarding the relationship between step rate and maximum velocity in trained sprinters [5-8]. Finally, mean step lengths of 2.24±0.16-2.14±0.14 m and 2.11±0.16-1.94±0.09 m have been reported for male and female sprinters, respectively, with a significant positive relationship between step length and maximum velocity [7,9,10]. Because these parameters are critical components of maximal velocity in practice, establishing normative data for elite males and females for these variables and understanding their relationship to sprint performance as measured by sprint time can give coaches valuable guidance for designing training plans.

In addition, lower body joint angular kinematics and foot touchdown velocities may be important determinants of maximal sprint velocity. Prior research has indicated that larger upper leg angular velocity and smaller upper leg angle at touchdown are related to increased maximal velocity [11-13]. Furthermore, horizontal foot velocity at touchdown has been correlated with sprint velocity in trained sprinters, though limited research exists on the importance of this variable [12,14]. Expanding the data set on these variables in an elite population and exploring their relationships to maximum sprint velocity may be important.

**Figure 1:**
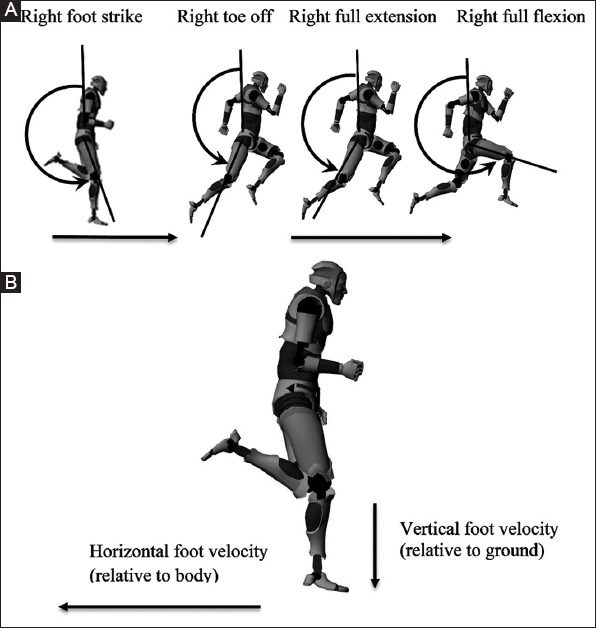
(A) Upper leg angles. (B) Foot velocity at touchdown.

While normative data for trained sprinters have been previously reported, it is currently unclear which of these variables are most important in a homogenous population of international-level sprinters. Therefore, in this investigation, we collected and analyzed anthropometric data and maximum velocity kinematics from practice of elite US 100 m sprinters. The purpose of this analysis was to: (a) Identify which anthropometric and kinematic variables were the strongest predictors of practice velocity when the group was separated by gender and (b) establish normative data sets for this unique population of elite sprinters. Based on prior research, our hypothesis was that step length, contact time, maximum upper leg extension angle (minimum angle relative to vertical, as shown in Figure 1A), and backward horizontal foot velocity at touchdown would be significantly correlated with maximum velocity in practice within the subsets of males and females [6,7,12-14].

## 2. Methods

### 2.1. Participants

A total of 38 (male=19, female=19, age=27.29±2.25 years) internationally competitive US 100 m sprinters participated in this study (mass: 69.32±10.04 kg; height: 1.73±0.08 m; BMI: 23.09±2.27 kg/m^2^). Participants were included if they made the 100 m final or semifinal in the US Outdoor National Championships during the 2015-2019 time period because this is the most recent data to indicate elite US 100 m sprint status (average 100 m race time: Males: 10.13±0.21 s, females 11.28±0.24 s) [15]. All participants signed and consented to an informed consent as part of the USA Track and Field high-performance program in accordance with the Declaration of Helsinki.

### 2.2. Data collection procedures

For the anthropometric variables, height and weight were self-reported by the athlete and leg length was determined by measuring from the greater trochanter to the ground (the track) for the right leg with a standard tape measure while the athlete was in running shoes. Next, participants completed a self-selected dynamic warm-up. This typically included various dynamic stretches, sprint drills, and submaximal sprints. For the maximal velocity sprint testing in practice, participants completed between two and five maximal effort sprint trials of 40-60 m in distance. Participants were instructed to self-select an acceleration distance of at least 30 m that would allow for the attainment of top speed before entering the video camera field of view, which was marked by cones on the track. Participants were also specifically instructed to maintain this top speed past the end of the field of view. The trial with the highest top speed (as determined by video, see below) was included in the statistical analysis.

**Figure 2:**
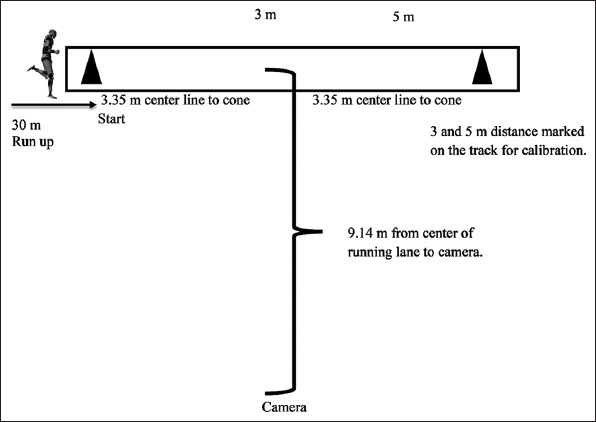
Data collection procedures

High-speed video (Casio EXILIM Pro EX-F1, 300 Hz, 1/1600, Tokyo, Japan) was used to collect the sagittal plane kinematic variables listed in Table 1. The video camera was positioned 9.14 m perpendicular to the center of the running lane, with the camera placed on a tripod at a height of approximately 1.0 m. The field of view was 6.7 m total, long enough to capture one complete stride cycle (two complete steps) for even the tallest and fastest sprinters. In the running lane, cones were placed at 3.0 and 5.0 m from the beginning of the field of view, to provide a static reference frame. A diagram of the data collection set-up is presented in Figure 2.

**Table 1:** Anthropometric, practice kinematic, and spatiotemporal data

Variable (mean±SD)	Males (*n*=19)	Females (*n*=19)
Leg length (m)	0.94±0.02	0.87±0.03
Velocity (m/s)	10.59±0.44	9.58±0.27
Step length (m)	2.25±0.11*	2.08±0.11**
Step length/leg length	2.39±0.11*	2.38±0.14**
Step rate (steps/s)	4.71±0.17	4.62±0.19
Contact time (s)	0.090±0.006	0.095±0.005
Flight time (s)	0.124±0.010	0.123±0.007
Upper leg full extension angle (deg)	154.53±5.16*	152.11±6.18
Upper leg full flexion angle (deg)	256.37±5.22	253.47±4.44
Time upper leg full flexion to touchdown (s)	0.11±0.01	0.11±0.01
Upper leg angle at touchdown (deg)	209.76±3.17	213.32±3.36
Total upper leg range of motion (deg)	101.92±6.24*	101.53±6.10
Upper leg angle at takeoff (deg)	155.53±3.81	154.66±5.21
Upper leg angular velocity (deg/s)	479.33±30.00*	468.82±26.15
Horizontal backward foot velocity at touchdown (m/s)	8.22±0.46*	7.32±0.39
Vertical foot velocity at touchdown (m/s)	2.20±0.37	2.03±0.37
Upper leg angular velocity peak flexion to touchdown (m/s)	418.10±32.56	382.67±47.38

* Significantly correlated with velocity in males (*P*<0.05).

** Significantly correlated with velocity in females (*P*<0.05).

The video was manually digitized and analyzed by one of three investigators using a proprietary kinematic software program [16]. The field of view in the running lane was calibrated using the 5 m mark as a static reference frame to create a two-dimensional (sagittal plane) scale within the CompuSport software program. All kinematic measures were determined for both the right and left legs during one full stride cycle in the field of view, and then, a trial average was calculated from the right and left leg values.

Kinematic variables included sprinting velocity, contact time, flight time, step rate, and step length. Contact time was determined as the instant of initial ground contact until the instant of takeoff on the same foot, as seen visually at 300 fps. Flight time was counted from the instant of takeoff on one foot until the instant of initial ground contact on the contralateral foot. Step rate was defined as the number of complete steps taken per second, calculated as the inverse of step time (contact time + flight time^-1^). Step length was defined as the horizontal displacement, calculated through digitized data, between successive ground contacts, as marked by the foremost part of the ground contact foot at initial touchdown. Sprinting velocity was calculated from the product of step rate and step length [7,17].

In addition, measures of upper leg (thigh segment) kinematics and foot touchdown velocities were determined. The upper leg variables were measured in a reference frame relative to the vertical counterclockwise to the thigh (illustrated in Figure 1A) and reported in units of degrees. These included upper leg peak extension angle (behind the body), upper leg peak flexion angle (high knee position in front of the body), upper leg full range of motion from peak extension to peak flexion, upper leg angle at touchdown, and upper leg angle at takeoff. Average upper leg angular velocity throughout the gait cycle was calculated from the upper leg full range of motion divided by the step time, and average upper leg angular velocity from peak flexion to touchdown was calculated by subtracting the upper leg angle at touchdown from the upper leg angle at peak flexion and dividing by the time required to complete this movement. The vertical component of foot velocity at touchdown was calculated relative to the ground, and horizontal backward foot velocity at touchdown was determined in a reference frame relative to the runner’s center of mass, as calculated by digitizing the runner’s body at touchdown. These variables were measured using the lateral or medial malleolus as it approached and made contact with the ground. Foot velocity variables are illustrated in Figure 1B [11,12,14].

### 2.3. Statistical analysis

The data were tested for normality by checking for skewness and kurtosis followed by the Kolmogorov–Smirnov test. Correlations between all the variables were assessed using either the Pearson’s or Spearman correlation coefficients depending on their distribution. With horizontal velocity as the dependent variable, preliminary analyses identified the following independent variables of interest: Step length, maximum upper leg extension angle (behind the body), and horizontal backward foot velocity at touchdown. These variables were selected based on a significant correlation with velocity. Independent variables showing high levels of collinearity were eliminated from consideration in the regression model. For males, a multiple regression was conducted for the variables that were significantly correlated with velocity in unadjusted analysis: For step length, maximum upper leg extension angle, and horizontal backward foot velocity at touchdown. Data are reported as mean±standard deviation. The *a priori* threshold for significance was set at α=0.05. All statistics were completed using Microsoft Excel and IBM SPSS Statistics for Windows, version 24 (IBM Corp., Armonk, N.Y., USA).

## 3. Results

All descriptive data are presented in Table 1. All results reported here are from the pairwise analysis unless stated otherwise.

### 3.1. Males

Step length (*r*=0.668; *P*=0.002), horizontal backward foot velocity at touchdown (*r*=0.459; *P*=0.048), and upper leg full extension angle (behind the body) (*r*=–0.585; *P*=0.009) were significantly correlated with practice velocity in males. Contact time (*r*=–0.424; *P*=0.071) was not significantly correlated with practice velocity in these elite male sprinters. An overall multiple regression model in the prediction of practice velocity (y) was fit including step length, upper leg full extension angle (behind the body), and horizontal backward foot velocity at touchdown. This multiple regression model showed that upper leg full extension angle and horizontal backward foot velocity at touchdown were no longer significant when taking account the other variables in the model. Similarly, a multiple regression model was fit for step length and upper leg full extension angle, and upper leg full extension angle was not significant in this model. A final multiple regression model in the prediction of practice velocity (y) was fit for step length and horizontal backward foot velocity at touchdown was not significant in this model. Because of these results, the most parsimonious final overall linear regression model in the prediction of practice velocity (y) contained only step length (Table 2). This model suggested that step length accounted for 44.6% of the variability in practice velocity in males.

**Table 2:** Coefficients for the dependent variable: Velocity (m/s)

Gender	Model	Unstandardized coefficients		t	Sig.

		B	Sth. error		
Female	(Constant)	6.357	0.967	6.576	0.000
	Step length (m)	1.551	0.465	3.335	0.004
Male	(Constant)	4.405	1.673	2.632	0.017
	Step length (m)	2.749	0.743	3.701	0.002

### 3.2. Females

Step length (*r*=0.629; *P*=0.004) was the only variable in this study that was significantly correlated with practice velocity in females. Contact time (*r*=–0.157; *P*=0.520), horizontal backward foot velocity at touchdown *(r*=–0.041; *P*=0.869), and upper leg full extension angle (*r*=-0.120; *P*=0.626) were not significantly correlated with practice velocity in females. A linear regression model suggested that step length accounted for 39.5% of the variability in practice velocity in females (Table 2).

## 4. Discussion

The purpose of this study was to analyze anthropometrics and kinematic variables at maximum velocity in practice in 100 m finalists or semifinalists in the 2015-2019 US National Championships. We aimed to identify the strongest predictors of practice velocity when the group was separated by gender and to establish normative data sets for this unique population of elite sprinters. The most important findings of this study were that step length, horizontal backward foot velocity at touchdown, and upper leg full extension angle were the strongest predictors of maximum velocity in practice in male elite US 100 m sprinters and step length was the strongest predictor of practice velocity in female elite US 100 m sprinters.

The practice velocity values in this study were on the upper end of those previously reported in the literature (10.84±0.12-9.33±0.31 m/s and 9.73±0.35-8.88±0.11 m/s) [5-7]. Contact time and flight time were both in the ranges previously reported (0.08-0.10 s and 0.11-0.13 s) [6,7]. Prior research has indicated that no significant relationship exists between flight time and velocity and there is a significant inverse relationship between contact time and velocity when the genders were separated, contact time was not significantly correlated with velocity which is in conflict with those studies [7,10]. These differences are attributed to the research population of non-elite sprinters. Step length and step rate values were higher than what the literature currently details, which may be because of the elite level of athletes in this study [7,10]. Prior research suggested a direct relationship between step length and velocity as we found in this investigation [7,10]. The previous studies indicated that horizontal backward foot velocity at touchdown was related to velocity, which supports our results [12].

This research contributes to the body of knowledge and benefits athletes, coaches, and scientists in a number of ways. First, this research provides a robust database for this elite subset that coaches and athletes can use for performance comparisons. This research establishes normative values for a wide range of practice variables for male and female international 100 m US sprinters, which before this did not exist in the literature, so this is a novel finding. This investigation pinpointed three variables in males, step length, horizontal backward foot velocity at touchdown, and upper leg full extension angle and one variable in females, step length, as the variables that have the strongest correlation to practice velocity. These findings provide coaches of elite sprinters important insight on where they need to focus training and differentiate the focus of coaches when dealing with males or females.

The convenience sampling method is a potential limitation of this study. Another potential limitation is that the subjects were aware they were being filmed as part of the USA Track and Field high-performance program and accompanying technical model, and it is unknown the extent to which this may have affected the sprint mechanics that were displayed during the data collection. In addition, confounding factors such as strength and time of year were not taken into account. Further research is warranted to clarify how the parameters evaluated here in practice environments translate into performance in competition. In addition, a deeper evaluation of how these velocity-related parameters can contribute to practical application for coaches is needed.

## 5. Conclusion

This research established specific practice normative values for classification as an international-level US male and female 100 m sprinter. In males and females, step length was the strongest predictor of practice velocity. In males only, with the exception of step length, horizontal backward foot velocity at touchdown and upper leg full extension angle had the strongest correlation to velocity in practice. These results indicate that generating velocity in practice for international-level 100 m male sprinters is a combination of anthropometric and kinematic variables. These predictors are potential areas of focus for sports scientists, athletes, and coaches.
